# How Do We Ensure the Quality of the Public Health Workforce?

**Published:** 2005-03-15

**Authors:** Stephen B Thacker

**Affiliations:** Office of Workforce and Career Development, Centers for Disease Control and Prevention

The events of September 11, 2001, brought unprecedented attention to public health in the United States. The national response to these events included a large infusion of resources into the public health system that enhanced the capacity for the system to respond to terrorist threats and other public health emergencies. However, as illustrated by the emerging epidemics of obesity and diabetes in this country, a disproportionate burden of disease, death, and disability in this century will continue to be attributable to chronic disease. To address this burden effectively requires the development of a workforce with new skills in addition to maintenance of evolving traditional competencies. In 2002, the Institute of Medicine (IOM) published a report, *Who Will Keep the Public Healthy?*, that targeted the training needs of the public health workforce in this century (1). The IOM report included a recommendation for federal agencies to provide incentives for developing academic–practice partnerships.

Two contributions to this issue of *Preventing Chronic Disease* address this recommendation and offer excellent illustrations of the benefits of such partnerships. Franks et al also address a second IOM recommendation for developing curricula in emerging areas of public health practice; they share their work in social marketing, physical activity, and evidence-based public health (2). Bodzin et al address both recommendations as they develop competencies and curricula in genomics, a field that few people understand well but one that will probably redefine both clinical medicine and public health practice (3). These investigators recognize both the training needs resulting from new challenges in public health and the opportunities to adopt new strategies and technologies to reach public health workers who are thirsty for the knowledge and skills that will enable them to do their jobs effectively.

The need to identify, train, and support the public health workforce has been long recognized (4-6). Although the Centers for Disease Control and Prevention (CDC), along with several other organizations in and outside of government, acknowledge the workforce as the essential element in public health, support for training in public health has been inconsistent and poorly funded. The director of the CDC in 2001 declared that the first priority of the agency was to build the capacity of the public health workforce (7). Following an extended process during 2003 and 2004 that included input from outside partners and the public, the CDC formed the Office of Workforce and Career Development (OWCD) to address needs within the agency and nationally.

To identify the target population and determine its training needs, the CDC has supported efforts in government and academia to update and supplement previous assessments (8,9). The OWCD has a leadership role in environmental scanning of learning methods and of new needs and disciplines in public health, evaluation of competency-based training activities, and research and innovation in learning methods. The OWCD is conducting similar activities for the CDC workforce while working with partners to develop competencies and multiple methods to deliver training effectively. The office also serves as an innovator in approaches to targeted recruitment, leadership development, succession planning, and retention. Through this new office, the CDC will serve as a focal point for developing the national public health workforce, coordinating information, sharing cutting-edge learning tools, and advocating for more general recognition of the importance of training that is conducted and evaluated rigorously. An essential element of this national role will be to strengthen current academic, private, and public partnerships and establish new partnership networks in workforce development and learning.

The workforce is the engine that makes the public health system function. In the face of new challenges in terrorism, injury prevention, chronic disease, and emerging infections, public health researchers are identifying effective prevention tools, using new disciplines such as genomics and informatics. In the context of globalization and heightened expectations, the public health workforce is aging and understaffed. Still, this workforce must be trained appropriately and supplemented with new highly trained workers prepared to meet these challenges. Unfortunately, training continues to be underappreciated and poorly funded. It is our challenge to make workforce recruitment and development and succession planning respected and supported priorities at the CDC and in the nation. Innovative work, such as that presented in this issue, is important, but far more needs to done. For it is, indeed, our responsibility as public health workers to meet our assurance obligation and make the development of ourselves and our coworkers a national priority.

## Author Information

Corresponding Author: Stephen B. Thacker, MD, MSc, Director, Office of Workforce and Career Development, Centers for Disease Control and Prevention, Mail Stop E-94, Atlanta, GA 30333. Telephone: 404-498-6010. E-mail: SThacker@cdc.gov.

## References

[1] Gebbie KM, Rosenstock L, Hernandez LM (2003). Who will keep the public healthy? Educating public health professionals for the 21st century.

[2] Franks AL, Brownson RC, Bryant C, Brown KM, Hooker SP, Pluto DM (2005). Prevention research centers: contributions to updating the public health workforce through training. Prev Chronic Dis [serial online].

[3] Bodzin J, Kardia SLR, Goldenberg A, Raup SF, Bach JV, Citrin T (2005). Genomics and public health: development of Web-based training tools for increasing genomic awareness. Prev Chronic Dis [serial online].

[4] U.S. Department of Health and Human Services, U.S. Public Health Service (1997). The public health workforce: an agenda for the 21st century, a report of the Public Health Functions Project.

[5] Potter MA, Pistella CL, Fertman CI, Dato VM (2000). Needs assessment and a model agenda for training the public health workforce. Am J Public Health.

[6] Gebbie KM (1999). The public health workforce: key to public health infrastructure. Am J Public Health.

[7] Koplan JP Building infrastructure to protect the public's health: a public health training network broadcast sponsored by the Association of State and Territorial Health Officials in partnership with HHS, CDC, HRSA, and FDA.

[8] Centers for Disease Control and Prevention (1992). Leadership development survey of state health officers — United States, 1988. MMWR.

[9] Council of State and Territorial Epidemiologists (CSTE) (2004). 2004 national assessment of epidemiologic capacity: findings and recommendations.

